# Metformin versus metformin plus pioglitazone on gonadal and metabolic profiles in normal-weight women with polycystic ovary syndrome: a single-center, open-labeled prospective randomized controlled trial

**DOI:** 10.1186/s13048-024-01367-7

**Published:** 2024-02-19

**Authors:** Han Zhao, Jiaqi Zhang, Chuan Xing, Xiangyi Cheng, Bing He

**Affiliations:** 1 Department of Endocrinology, The Affiliated Wuxi People’s Hospital of Nanjing Medical University, Nanjing Medical University, Wuxi People’s Hospital, Wuxi Medical Center, Wuxi, Jiangsu 214000 PR China; 2 Department of Endocrinology, Shengjing Hospital, China Medical University, Shenyang, Liaoning 110000 PR China

**Keywords:** Polycystic ovary syndrome, Normal-weight, Metformin, Pioglitazone, Randomized controlled trial

## Abstract

**Objective:**

To investigate the effects of metformin (MET) monotherapy and pioglitazone plus MET (PIOMET) therapy on gonadal and metabolic profiles in normal-weight women with polycystic ovary syndrome (PCOS).

**Methods:**

Sixty normal-weight women with PCOS were recruited between January and September 2022 at the Shengjing Hospital of China Medical University. They were randomly assigned to the MET or PIOMET groups for 12 weeks of MET monotherapy or PIOMET therapy. Anthropometric measurements, menstrual cycle changes, gonadal profiles, and the oral glucose insulin-releasing test (OGIRT) were performed at baseline and after the 12-week treatment.

**Results:**

Thirty-six participants completed the trial. MET and PIOMET therapies improved menstrual cycles after the 4- and 12-week treatments; however, there was no statistical difference between the two groups. PIOMET therapy improved luteinizing hormone (LH), luteinizing hormone/follicle stimulating hormone (LH/FSH) ratio, and free androgen index (FAI) levels after the 4-week treatment, whereas MET monotherapy only improved total testosterone (TT) levels compared to baseline (*P* < 0.05). Both MET and PIOMET therapies improved TT and anti-Mullerian hormone (AMH) levels after the 12-week treatment (*P* < 0.05). In addition, only PIOMET therapy significantly improved sex hormone-binding globulin (SHBG), FAI, and androstenedione (AND) levels than the baseline (*P* < 0.05). PIOMET therapy improved SHBG and AMH levels more effectively than MET monotherapy (*P* < 0.05). Furthermore, PIOMET treatment was more effective in improving blood glucose levels at 120 and 180 min of OGIRT compared to MET monotherapy (*P* < 0.05).

**Conclusions:**

In normal-weight women with PCOS, PIOMET treatment may have more benefits in improving SHBG, AMH, and postprandial glucose levels than MET monotherapy, and did not affect weight. However, the study findings need to be confirmed in PCOS study populations with larger sample sizes.

## Introduction

Polycystic ovary syndrome (PCOS) is a common reproductive endocrine and metabolic disease among women of childbearing age and is the most common cause of anovulation infertility [1], with a prevalence rate of approximately 10‒15% [2]. Its significant features include hyperandrogenism, chronic anovulation, and polycystic ovaries, often accompanied by abdominal obesity, insulin resistance (IR), dyslipidemia, and other metabolic disorders [3]. Clinically, although most PCOS cases occur in obese/overweight women, 25% of women still have a normal body mass index (BMI) [4]. IR is one of the pathogeneses of PCOS, which can aggravate hyperandrogenemia (HA) in women with PCOS, affect their reproductive function, and increase the risk of metabolic diseases. Previous studies have found that the prevalence of IR in overweight and obese women with PCOS is up to 95%, whereas that in normal-weight women is up to 75% [5]. In addition, normal-weight women with PCOS had higher fasting blood glucose levels [6] and an increased risk of prediabetes, type 2 diabetes (T2DM), and gestational diabetes than BMI-matched non-PCOS women [7–10]. Therefore, IR, an inherent characteristic of the PCOS population [4, 11, 12], should be managed as early as possible, regardless of BMI.

Clinically, there are many therapeutic options for overweight and obese women with PCOS, such as metformin (MET), glucagon-like peptide 1 receptor agonist, and orlistat, which can reduce weight, improve IR, and thus improve hyperandrogenemia and ovulation disorder. However, clinical options for normal-weight women with PCOS are limited. Thiazolidinediones (TZDs) are peroxisomal proliferating-activated receptor-γ (PPAR-γ) agonists, such as pioglitazone and troglitazone, which play an insulin-sensitizing role by stimulating PPAR-γ receptors in peripheral tissues [13, 14]. TZDs can alleviate HA by upregulating the expression of liver sex hormone-binding globulin (SHBG), directly regulate ovarian function through a variety of mechanisms [15, 16], and play a role in preventing the metabolic consequences in PCOS women [17–20]. Previous meta-analysis suggest that pioglitazone (PIO) is better than MET in improving the menstrual cycle and ovulation in women with PCOS. However, because of the side effects of water and sodium storage in TZDs, weight gain can be observed after PIO treatment, which limits its application in some obese women with PCOS [21]. Animal studies have shown that PIO plus MET (PIOMET) synergistically improves HA and follicular morphology in obese PCOS rats without changing their body weight [17]. Moreover, small-sample studies found that although MET and rosiglitazone did not affect body weight in obese and non-obese women with PCOS, there was no significant difference between the combined treatment and monotherapy [22, 23]. Therefore, we carefully designed this randomized controlled trial to study the effect of PIOMET compared to MET alone in the normal-weight PCOS population, aiming to explore more suitable clinical treatment schemes for this group of patients.

## Methods

### Participants

From January to September 2022, we enrolled 60 women diagnosed with PCOS in the outpatient Department of Endocrinology at Shengjing Hospital of China Medical University. This single-center, open-label, 1:1 randomized controlled trial was approved by the Research and New Technology Ethics Committee of Shengjing Hospital, China Medical University (No.2022PS671K) and pre-registered in ClinicalTrials (registration number: NCT05519813). All patients were informed of the purpose of the study and signed a written informed consent form prior to participation.

### Inclusion and exclusion criteria

Inclusion criteria were as follows: (i) 18‒40 years of age; (ii) 18.5 ≤ BMI < 25 kg/m^2^; (iii) diagnosis of PCOS meeting the Rotterdam 2003 criteria; and (iv) phenotype B with HA and oligovulation/anovulation [24].

The exclusion criteria were as follows: (i) pregnant patients or patients who intended to become pregnant, were breastfeeding, or did not consent to contraception; (ii) patients on medications that affected insulin sensitivity or ovarian function during the last 3 months; (iii) comorbidity (diabetes mellitus, thyroid dysfunction, hyperthyroidism or hypothyroidism, 21-hydroxylase deficiency, hyperprolactinemia, androgen-secreting neoplasms, congenital adrenal hyperplasia, and Cushing’s syndrome) (all based on the patient’s medical history); (iv) severe liver (alanine aminotransferase, aspartate aminotransferase > 2 times normal) or renal function (estimated glomerular filtration rate, eGFR < 60 mL/min/1.73 m^2^) damage; (v) current or past (last 3 months) participation in other interventional studies; (vi) 17-hydroxyprogesterone levels > 2 ng/mL (excluding women with HA due to atypical 21-hydroxylase deficiency); and (vii) women who were allergic to PIO or MET, had severe cardiovascular disease and gastrointestinal problems, a history of cancer, and had active infections or other conditions that may endanger patient safety.

### Learning plan

This was a prospective, randomized, open-label, parallel-group, controlled trial. Eligible patients with PCOS were recruited and randomly assigned to the PIOMET or MET groups after obtaining informed consent. Randomization was performed using computer-generated sequences of random numbers. PIOMET and MET tablets were provided by HUADONE MEDICINE (Hangzhou, China) and the Bristol-Myers Squibb Company (Shanghai, China), respectively. The patients were required to take 2 tablets/day for both PIOMET (each tablet containing 15 mg of PIO and 500 mg of MET) and MET (500 mg/tablet). All eligible patients were instructed to maintain their normal diet, exercise, and contraceptive use throughout the study. They were also asked to abstain from medications that may have endocrine or metabolic effects.

Each participant completed the assessment at three time points: baseline and 4 and 12 weeks after randomization. All patients with PCOS fasted at the time of measurement. Data on body composition, menstrual cycle, glucose homeostasis, and sex steroid hormone concentration were measured and recorded at the beginning of the study. Only sex steroid hormones were measured after 4 weeks of treatment, and all basal assessments were repeated at the end of the study. Patients with PCOS were frequently contacted weekly by telephone or other communication means to inquire about their body weight, menstrual cycles, and adverse drug reactions; remind them to take their medication daily; and arrange convenient times for their next visit.

### Evaluation of BMI

Anthropometric data, including height, weight, and BMI, were obtained using standardized protocols. The height and weight of each participant (wearing light indoor clothing without shoes) were measured and recorded by a nurse, and the BMI [weight (kg)/ height (m)^2^] was calculated. We defined normal weight as 18.5 ≤ BMI < 25 kg/m^2^, according to the World Health Organization [25]. Height and body weight were measured using a standardized wall-mounted radiometer (± 0.1 cm) (SECA 71; Hamburg, Germany), and multifrequency bioelectrical impedance analyzer (Inbody770 scanner; the In-Body Bldg. Seoul, Korea), respectively.

### Menstruation assessment

All participants met the Rotterdam 2003 criteria for phenotype B [24], with ovulation dysfunction (oligoovulation/anovulation) and menstrual cycle disorders, including sporadic menses and amenorrhea. Menstrual regularity at the time of enrollment was assessed using self-reported menstrual intervals of the past 3 years, which were based on a diary review. A sporadic menstrual cycle was defined as having fewer than six periods in 12 months, while amenorrhea was defined as cessation of menstruation for more than 6 months. Each bleeding count was performed during one menstrual cycle. Menstrual cycle recovery was defined as recurrence of a patient’s normal menstrual cycle. During the 12 weeks of treatment, the patients were asked to use only barrier contraception, and after 12 weeks, menstrual cycle changes were recorded.

### Evaluation of biochemical parameters

The levels of follicle-stimulating hormone (FSH) (mIU/mL) and luteinizing hormone (LH) (mIU/mL) were measured using chemiluminescence immunoassay. Total testosterone (TT) (ng/mL) was determined using an electrochemiluminescence immunoassay (ECLLA). HA was defined as a TT higher than 0.5 ng/mL [26]. Immuno-chemiluminescence (Unicel DXL 800; Beckman Coulter, USA) was used to detect SHBG (nmol/L). TT (ng/mL) was converted to TT (nmol/L) by multiplying it by 3.467. Free androgen index (FAI) was calculated as follows = TT (nmol/L)/SHBG (nmol/L) ×100 [27]. The levels of androstenedione (AND) (ng/mL), dehydroepiandrosterone sulfate (DHEAS) (ng/mL), and anti-Mullerian hormone (AMH) (ng/mL) were measured using the luminescence method. LH, FSH, TT, SHBG, and FAI levels were assessed at baseline and at 4 and 12 weeks after randomization. AMH, AND, and DHEAS levels were assessed at baseline and 12 weeks after randomization.

We performed a 75-g oral glucose insulin-releasing test (OGIRT) at baseline and after 12 weeks of random group allocation to assess glucose tolerance and insulin sensitivity.The participants fasted for 8–12 h overnight, and venous blood samples were collected at 0, 60, 120, and 180 min after the glucose meal. Plasma glucose (mmol/L) and insulin (mU/L) levels were measured using the standard glucose oxidase method and Abbott CI16200 radioimmunoassay. The homeostasis model assessment of insulin resistance (HOMA-IR) score was calculated to assess IR. HOMA-IR score = fasting insulin (FINS) (mU/L) × fasting plasma glucose (FPG) (mmol/L)/22.5 [28]. The area under the glucose curve (AUCGlu) (mmol/L·min) and the area under the insulin curve (AUCIns) (mU/L·min) were obtained by calculating the sum of the trapezoidal areas at 0, 60, 120, and 180 min, and the ratio of the two curves, AUCIns/AUCGlu, was calculated.

### Sample size estimation

The sample size of the study was considered, calculated, and analyzed comprehensively. Considering that the primary outcome was changes in sex steroid hormones after 12 weeks, the sample size calculation strategy was based on the assumption of a mean SHBG addition in the MET group [25.40 (19.60–39.80)] and an expected SHBG addition of more than twofold in the PIOMET group [53.70 (35.53–73.60)]. Thus, we required 16 participants in each group. Forty patients were examined and equally assigned to each group (*N* = 20) according to α = 0.05, power = 80%, and loss to follow-up of approximately 20%.

### Statistical analysis

Continuous data were presented as mean ± SD or median (25th–75th percentile). Categorical data were presented as frequencies or percentages. First, the normality of continuous data was assessed using the Pearson synthesis/Shapiro–Wilk test. A paired t-test (normal distribution) or paired Wilcoxon test (non-normal distribution) was used for intragroup comparisons of the continuous data. Furthermore, independent-sample t-tests (normally distributed) or Mann–Whitney U tests (not normally distributed) were used to compare the groups. Categorical variables were expressed as frequencies or percentages and compared using the chi-square test. Statistical significance was set at *P* < 0.05 (two-tailed). All data analyses were performed using GraphPad Prism 9.0.1 (GraphPad Software, Chicago, IL, USA) and SPSS 27.0 (SPSS Inc., Chicago, IL, USA).

## Results

### Participants

**Fig. 1:**
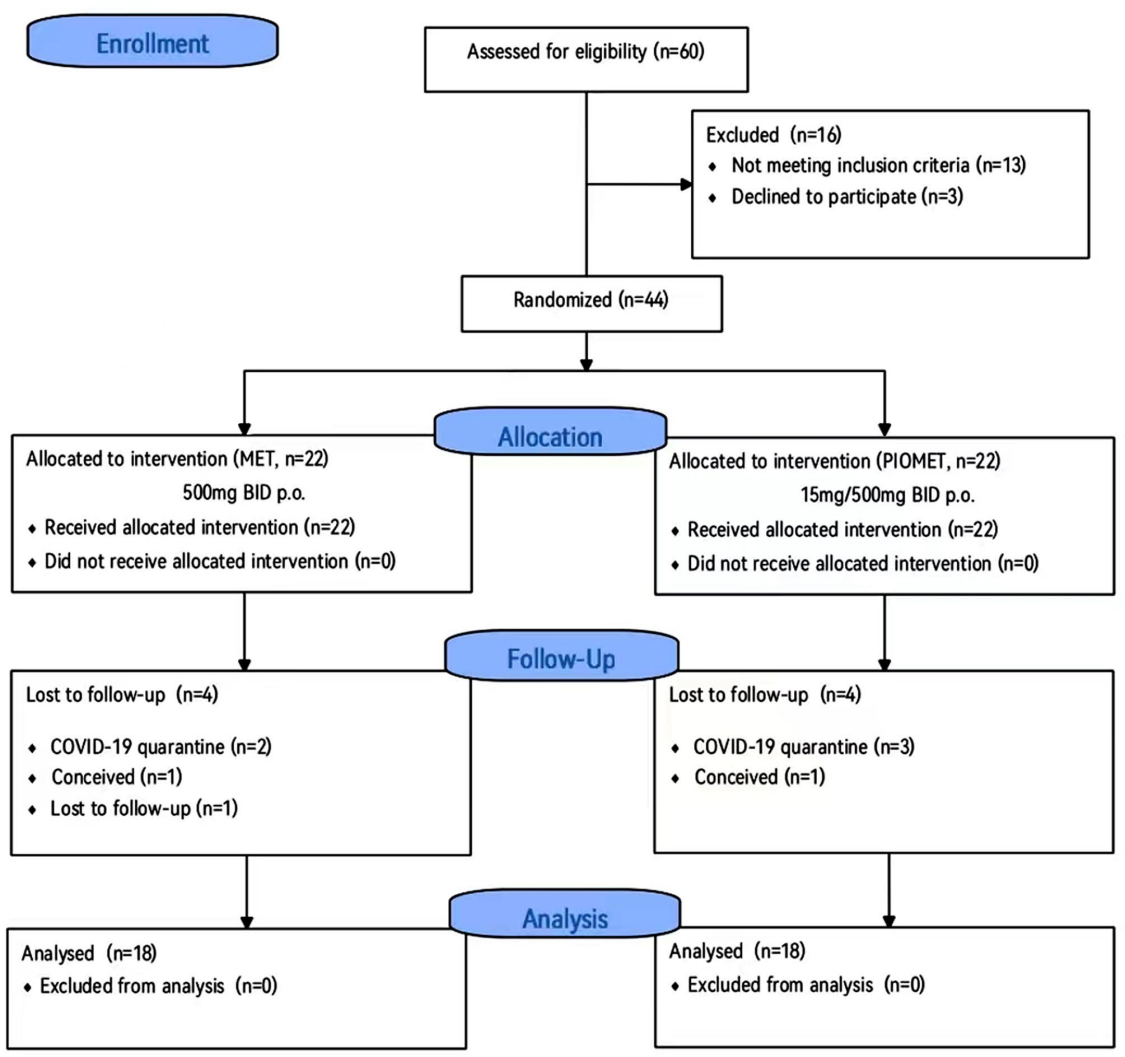
Patient selection flow diagram

Sixty patients with PCOS were recruited from the outpatient endocrinology department according to the 2003 Rotterdam criteria. During the screening, 16 patients were excluded for apparent reasons: six intended to become pregnant during the trial, five patients had a history of multiple contraceptive uses, three patients refused to participate, and two patients had comorbidities (hypothyroidism). Subsequently, 44 patients with PCOS who met the inclusion criteria were enrolled in the study, including 22 in the PIOMET group and 22 in the MET group. Four patients in the PIOMET group dropped out (one had an unintended pregnancy and the other three were lost to follow-up due to poor compliance and busy working schedules). Four patients in the MET group dropped out of the trial (two patients were lost to follow-up, one was affected by COVID-19 isolation, and one had an unintended pregnancy). Finally, 18 participants each in the PIOMET group and 18 in the MET group who completed the trial were included in the final analysis. The follow-up rate for both the MET and PIOMET groups was 81.82% (18/22) (Fig. 1).

### Baseline information

Anthropometric parameters (age, height, weight, and BMI), gonadal parameters (LH, FSH, LH/FSH, TT, SHBG, FAI, AND, DHEAS, and AMH), and metabolic parameters (FPG, FINS, HOMA-IR, AUCGlu, AUCIns, and AUCGlu/AUCIns) were measured and showed no significant differences at baseline (*P* > 0.05). All the participants were oligomenorrheic. The baseline data are presented in Table 1.

**Table 1:** Demographic data and baseline characteristics of patients

	PIOMET (*N* = 18)	MET (*N* = 18)	P value
Age (years)	25.33 ± 5.45	23.94 ± 5.29	0.4428
Height (m)	1.64 ± 0.05	1.64 ± 0.06	0.9138
Body weight (kg)	57.22 ± 6.24	58.69 ± 6.72	0.5001
BMI (kg/m^2^)	21.63 (19.99–23.37)	23.32 (19.84–23.84)	0.3040
AMH (ng/mL)	13.53 ± 5.54	10.25 ± 6.13	0.1013
LH (mIU/mL)	16.45 (13.15–22.26)	15.26 (11.81–22.31)	0.5841
FSH (mIU/mL)	7.02 ± 1.87	7.28 ± 2.39	0.7279
LH/FSH	2.83 ± 1.41	2.57 ± 1.34	0.5791
TT (ng/mL)	0.74 (0.59–0.92)	0.79 (0.63–0.99)	0.5572
FAI (%)	6.45 (4.37–10.56)	13.62 (7.07–18.62)	0.0616
SHBG (nmol/L)	41.25 (30.55–54.20)	26.10 (14.73–38.35)	0.1038
AND (ng/mL)	4.21 ± 1.68	3.96 ± 2.34	0.7532
DHEAS (ng/mL)	276.80 ± 103.80	260.80 ± 84.56	0.6661
FPG (mmol/L)	5.21 (4.99–5.57)	5.31 (4.79–5.44)	0.7260
FINS (mU/L)	6.90 (5.75–11.85)	10.05 (5.60–13.50)	0.5960
HOMA-IR	1.79 (1.31–2.71)	2.46 (1.29–3.15)	0.4828
AUCIns (mU/L*min)	6615 (4853–11,156)	10,872 (6875–17,778)	0.1061
AUCGlu (mmol/L*min)	1162 (930.5–1371)	1213 (997.8–1335)	0.7084
AUCIns/AUCGlu	6.99 ± 3.91	11.13 ± 7.78	0.0584

PIOMET, pioglitazone hydrochloride and metformin hydrochloride tablets; MET, metformin; BMI, body mass index; FSH, follicle stimulating hormone; LH, luteinizing hormone; TT, total testosterone; FAI, free androgen index; SHBG, sex hormone-binding globulin; AND, androstenedione; FPG, fasting plasma glucose; FINS, fasting insulin; AUCGlu, area under the glucose curve; AUCIns, area under the insulin curve; HOMA-IR, homeostasis model assessment-insulin resistance; Results are expressed as mean ± SD or median (25th–75th percentile)

### BMI

After 12 weeks of treatment, weight loss was significant in the MET group (*P* = 0.0204), whereas it did not change in the PIOMET group. There was no significant difference in BMI between the two groups, and no significant differences in the improvement of body weight and BMI were observed between the two groups (Table 2).

**Table 2:** Information of 12-weeks post treatment and changes in endocrine and metabolic profile

	PIOMET (*N* = 18)		MET (*N* = 18)		*P* value (Change)
	12 weeks	Change from baseline	12 weeks	Change from baseline	
Anthropometric characteristics					
Body weight (kg)	57.00 ± 5.85	0.00 (-0.13 to 0.00)	58.56 ± 6.64 *	0.00 (-0.05 to 0.00)	0.7318
BMI (kg/m^2^)	21.63 (19.84–23.20)	0.00 (-0.05 to 0.00)	23.22 (19.71–23.76)	0.00 (-0.17 to 0.00)	0.8021
Gonadal hormones					
AMH (ng/mL)	10.36 ± 4.14 *	-2.79 (-4.68 to -1.35)	9.60 ± 5.11*	-0.74 (-1.67 to -0.01)	**0.0017**
LH (mIU/mL)	15.68 (10.15–21.75)	-2.76 (-6.32 to 6.33)	10.20 (6.39–16.23) *	-4.22 (-16.33 to 0.25)	0.4186
FSH (mIU/mL)	7.37 ± 2.11	0.31 (-1.15 to 2.24)	5.51 ± 2.05	-0.94 (-3.01 to 0.38)	0.0677
LH/FSH	2.20 ± 1.18	-0.42 (-1.24 to 0.10)	2.11 ± 0.95	-0.16 (-1.91 to 0.64)	0.8069
TT (ng/mL)	0.63 (0.44–0.73) *	-0.15 ± 0.21	0.77 (0.60–0.86) *	-0.10 ± 0.18	0.4154
FAI (%)	4.21 (1.99–7.12) *	-3.41 ± 5.98	11.67 (6.41–15.86)	-1.16 ± 5.30	0.2597
SHBG (nmol/L)	53.70 (35.53–73.60) **	14.50 (-1.45 to 31.33)	25.40 (19.60–39.80)	1.60 (-5.85 to 5.30)	**0.0150**
AND (ng/mL)	3.51 ± 1.60 *	-1.79 (-2.79 to -0.42)	3.46 ± 1.89	-0.79 ± 1.12	0.321
DHEAS (ng/mL)	274.20 ± 115.70	-17.74 ± 59.78	288.70 ± 109.40	23.96 ± 52.03	0.1013
Glucose and lipid-related parameters					
FPG (mmol/L)	5.21 (4.92–5.38)	-0.31 (-0.46 to 0.26)	5.25 (4.79–5.56)	-0.03 (-0.27 to 0.33)	0.3254
FINS (mU/L)	7.70 (4.90–9.30)	-1.38 ± 3.92	10.35 (5.95–14.75)	-0.71 ± 4.40	0.699
HOMA-IR	1.66 (1.16–2.16)	-0.47 ± 1.08	2.44 (1.24–3.62)	-0.20 ± 1.23	0.5762
AUCIns (mU/L*min)	5436 (3981–6612)	-1556 (-4410 to 500.3)	12,633 (7049–18,530)	780 (-4401 to 4317)	0.1693
AUCGlu (mmol/L*min)	1167 (839.6–1343)	-56.90 ± 236.20	1249 (1139–1515)	98.11 ± 330.90	0.2071
AUCIns/AUCGlu	5.12 ± 1.54	-1.80 ± 3.51	10.49 ± 4.97	-0.71 ± 3.44	0.4605

PIOMET, pioglitazone hydrochloride and metformin hydrochloride tablets; MET, metformin; BMI, body mass index; AMH, anti-Mullerian hormone;FSH, follicle-stimulating hormone; LH, luteinizing hormone; TT, total testosterone; FAI, free androgen index; SHBG sex hormone-binding globulin; AND, androstenedione; DHEAS, dehydroepiandrosterone sulfate; FPG, fasting plasma glucose; FINS, fasting insulin; AUCGlu, area under the glucose curve; AUCIns, area under the insulin curve; HOMA-IR, homeostasis model assessment-insulin resistance

The bold font indicates statistically significant between the two groups

* *P* < 0.05, vs. baseline and 12-week visits

** *P* < 0.01, vs. baseline and 12-week visits

### Menstrual status

After 12 weeks of treatment, menstrual cycle disorders improved in the PIOMET (*P* < 0.0001) and MET (*P* = 0.0002) groups. The menstrual cycle recovery rate was 66.67% (12/18) in the MET group and 88.89% (16/18) in the PIOMET group. There were no significant differences between the two groups (Table 2).

### Evaluation of gonadal parameters

After 4 weeks of treatment, the levels of LH (*P* = 0.0244), LH/FSH (*P* = 0.0266), and FAI (*P* = 0.0391) were significantly decreased in the PIOMET group compared to baseline, and only TT was significantly decreased in the MET group (*P* = 0.0203). The levels of LH (*P* = 0.0471) and LH/FSH (*P* = 0.0498) in the PIOMET group were significantly lower than those in the MET group, and there were no significant differences in the levels of FSH, TT, SHBG, and FAI between the two groups after treatment (Table 3).

**Table 3:** Information of 4-weeks post treatment and changes in gonadal hormones

Gonadal hormones	PIOMET (*N* = 18)		MET (*N* = 18)		*P* value (Change)
	4 weeks	Change from baseline	4 weeks	Change from baseline	
LH (mIU/mL)	6.30 (3.99–19.84) *	-13.10 ± 15.17	11.08 (7.25–21.17)	-2.99 ± 9.47	**0.0471**
FSH (mIU/mL)	6.32 ± 2.78	-1.19 ± 2.26	6.59 ± 1.94	-0.46 ± 2.60	0.4648
LH/FSH	1.76 ± 1.34 *	-1.42 ± 1.81	2.16 ± 1.25	-0.22 ± 1.14	**0.0498**
TT (ng/mL)	0.62 (0.44–0.77)	-0.17 (-0.24 to 0.08)	0.62 (0.55-1.00) *	-0.13 (-0.25 to -0.09)	0.9708
FAI (%)	4.85 (3.95–10.81) *	-1.03 (-7.27 to -0.09)	9.82 (6.63–16.52)	-1.49 (-4.86 to -0.51)	0.0596
SHBG (nmol/L)	42.70 (33.60–66.40)	8.49 ± 15.78	26.65 (15.88–37.30)	0.44 ± 5.74	0.881

PIOMET, Pioglitazone Hydrochloride and Metformin Hydrochloride Tablets; MET, metformin; FSH, follicle-stimulating hormone; LH, luteinizing hormone; TT, total testosterone; FAI, free androgen index; SHBG sex hormone-binding globulin. Results are expressed as mean ± SD or median (25th–75th percentile). The bold font indicates statistically significant between the two groups. * *P* < 0.05, vs. baseline and 4-week visits

At 12 weeks of intervention, AMH (*P* < 0.0001), TT (*P* = 0.0107), FAI (*P* = 0.0155), and AND (*P* = 0.0101) levels were significantly lower, whereas SHBG (*P* = 0.0039) levels were significantly higher than baseline in the PIOMET group. In the MET group, LH (*P* = 0.0435), AMH (*P* = 0.0202), and TT levels (*P* = 0.0466) significantly decreased compared to baseline. Compared to that in the MET group, the level of AMH in the PIOMET group was significantly decreased (*P* = 0.0017), and the level of SHBG was significantly increased (*P* = 0.0150). There were no significant differences in the levels of LH, FSH, LH/FSH, TT, FAI, AND, and DHEAS between the two groups (Table 2).

### Glucose homeostasis assessment

**Fig. 2:**
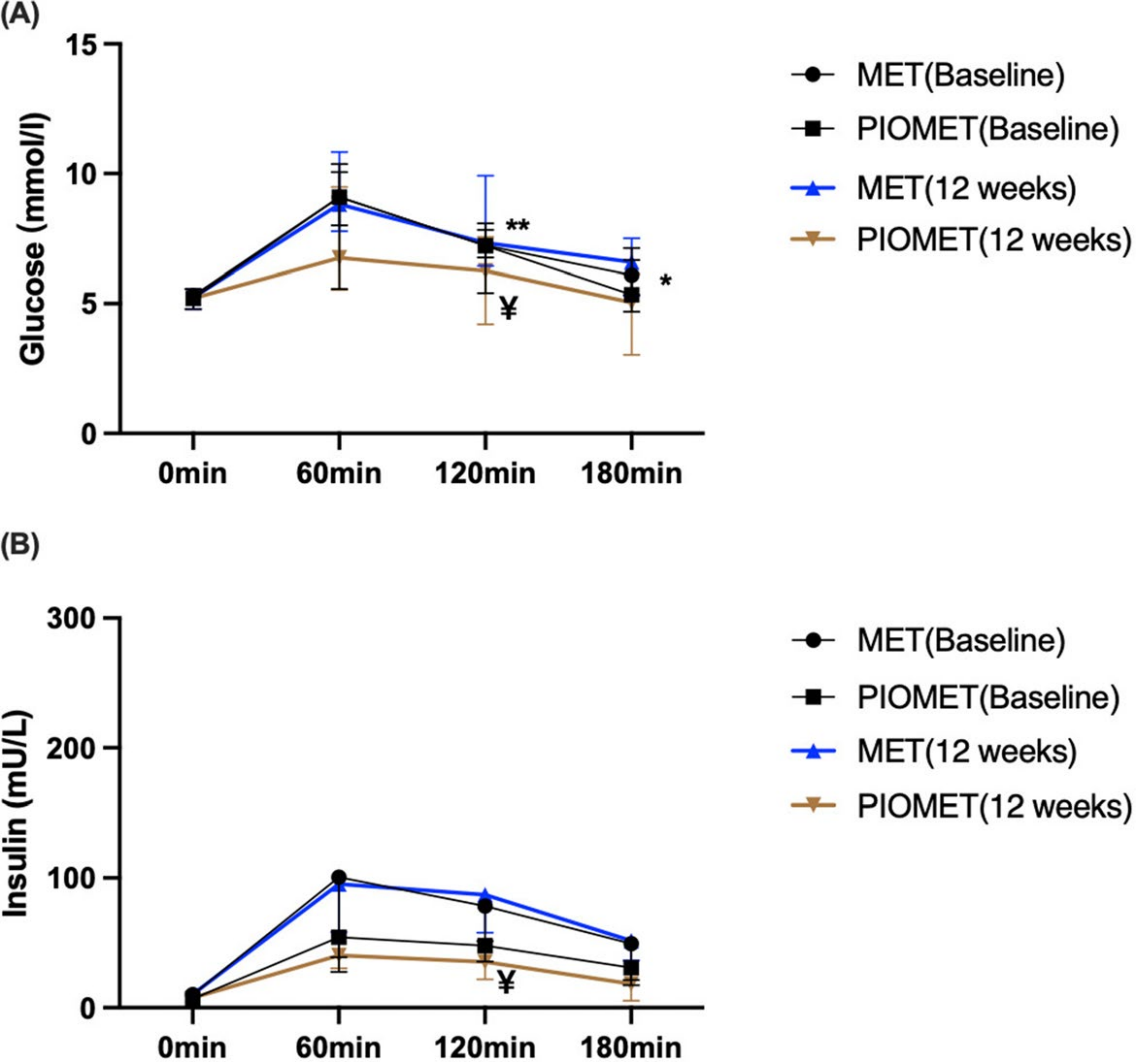
OGIRT after MET and PIOMET therapy

After the 12-week treatment, we compared the blood glucose and insulin levels after the OGIRT. The intra-group comparison showed that FPG, FINS, and HOMA-IR in both the PIOMET and MET groups decreased compared to baseline. However, these differences were not statistically significant (*P* > 0.05). In addition, AUCGlu, AUCIns, and AUCIns/AUCGlu did not improve in either group after the 12-week treatment (*P* > 0.05); however, they showed a decreasing trend in the PIOMET group (Fig. 2(A) and (B) and Table 2).

As shown in Fig. 2A and B, after the 12-week treatment in the PIOMET group, the OGIRT showed a downward trend in blood glucose and insulin levels at all time points. Compared to baseline, the PIOMET group showed a significant decrease in blood glucose (*P* = 0.0122) and insulin (*P* = 0.0093) levels at 120 min, whereas no significant changes were observed in the MET group (*P* > 0.05). Compared to the MET group, the PIOMET group showed a significant decline in blood glucose levels at 120 min (*P* = 0.0081) and 180 min (*P* = 0.0350) of the OGIRT after 12 weeks of treatment, and there was no significant difference at other time points (Fig. 2A and B).

### Adverse events

The treatment was generally well-tolerated. No serious adverse events were associated with MET or PIOMET therapy. One patient in the MET group had mild abdominal distension at the beginning of the treatment, but the symptom resolved after 2 weeks of therapy. All patients reported no headaches, nausea, vomiting, or gastrointestinal side effects. In the PIOMET group, no adverse events, including headaches, nausea, urinary tract infections, or lower limb edema, occurred during the treatment period.

## Discussion

The study was conducted as a randomized controlled trial to compare the effects of PIOMET therapy and MET monotherapy on menstrual cycles, sex hormones, and glucose metabolism in normal-weight patients with PCOS. Our results suggest that 12 weeks of PIOMET treatment significantly improved AMH, TT, SHBG, FAI, and AND levels in normal-weight women with PCOS, and PIOMET was more effective than MET in improving AMH and SHBG levels. In addition, after 12 weeks of treatment with PIOMET, glucose and insulin levels in the OGIRT at 120 min were significantly improved from baseline, and PIOMET was more effective than MET in improving blood glucose levels at 120 and 180 min of the OGIRT. We also found that PIOMET did not increase the weight of patients with PCOS during treatment and no adverse events occurred. Both the PIOMET and MET groups showed significant improvement in the menstrual cycle; however, there was no statistical difference between the two groups.

Menstrual disorders and anovulation are the main reasons why most women with PCOS of childbearing age seek medical treatment, and the recovery of menstruation and ovulation is their main goal [29]. Measurement of the menstrual cycle is often used as an accurate substitute index for oligoovulation in women of childbearing age in clinical practice, and up to 85% of women with PCOS show abnormal menstruation [30]. MET may have direct and indirect effects on the ovary by improving the activity of insulin- and steroid-producing enzymes [31]. The PPAR-γ gene is involved in regulating ovarian function, and TZDs also have a positive effect on the ovary, such as counteracting tumor necrosis factor-α inhibition of FSH-induced follicular development and steroidogenesis in an in vitro mouse preantral follicle culture system [29]. Previous studies have shown that MET and PIO can promote menstrual recovery in women with PCOS [29]. Tan et al. observed a shorter menstrual cycle in the rosiglitazone group after 6 months of MET therapy (1500 mg/day) versus rosiglitazone (4 mg/day) treatment in a normal-weight PCOS population (BMI < 25 kg/m^2^) [32]. Our meta-analysis showed that TZDs combined with MET improved the menstrual cycle in overweight and obese women with PCOS (BMI ≥ 25 kg/m^2^) significantly better than MET alone [33]. In normal-weight patients with PCOS, we also observed significant improvement in menstrual rates after 12 weeks of treatment in the MET (66.67%) and PIOMET (88.89%) groups, although there was no significant difference between the two groups. The small number of participants and short duration of the intervention can lead to heterogeneity. To date, few clinical studies have compared the effects of MET and PIOMET on menstrual changes in normal-weight patients with PCOS. Therefore, further studies are required to elucidate their effects.

HA is one of the characteristic changes in PCOS, and sustained high androgen levels can lead to follicular atresia [34]. In addition, the HA phenotype was found to be an independent predictor of T2DM in a subgroup of normal-weight women with PCOS [35]. MET effectively reduced testosterone levels in both normal and overweight women with PCOS [36]. Clinical trials in overweight and obese patients with PCOS (BMI ≥ 25 kg/m^2^) have shown that PIOMET (30‒45 mg/d for PIO and 1000‒1500 mg/d for MET) treatment for 3 months can effectively reduce testosterone levels compared to MET monotherapy (1000‒1500 mg/d) [37, 38]. In our previous meta-analysis of patients with PCOS without weight restriction, TZDs alone were not superior to MET in reducing testosterone levels. However, TZDs combined with MET significantly reduced TT levels compared with MET [14]. Similarly, we found significant reductions in TT levels after 4 and 12 weeks of MET treatment. In the PIOMET group, FAI decreased significantly at the beginning of 4 weeks, and TT, SHBG, and FAI levels significantly improved with the extension of treatment time, suggesting that PIO and MET may play a synergistic role in reducing hyperandrogenemia in normal-weight women with PCOS.

SHBG is a glycoprotein synthesized by the liver that can bind free testosterone with a high affinity, thus reflecting active testosterone in the body [39]. Concurrently, SHBG is also regarded as a clinical marker of metabolic abnormalities in PCOS. Low SHBG levels in women are strongly correlated with metabolic syndrome components (such as obesity, lipid profile, IR, and preDM) [40, 41] and are associated with the long-term prognosis of PCOS. Hyperinsulinaemia (HI) can inhibit the expression of SHBG in the liver, thus aggravating hyperandrogenemia [1]. A previous meta-analysis of overweight and obese patients with PCOS (BMI ≥ 25 kg/m^2^) showed that TZD combined with MET significantly increased serum SHBG levels compared to MET monotherapy [33]. Our animal experiments also showed that PIOMET significantly upregulated SHBG protein expression in the liver of PCOS rats compared to MET alone, which may be related to the upregulated expression of the upstream transcription factor, hepatocyte nuclear factor 4 of SHBG, by PIO [42]. Furthermore, the effect of PIOMET on SHBG was further corroborated by our clinical trial, where we observed a significant increase in SHBG in the PIOMET group after 12 weeks of treatment in normal-weight patients with PCOS, which significantly outperformed MET monotherapy. In addition, a significant downregulation of AND levels from baseline was also observed in the PIOMET group but not in the MET group.

PCOS in nonobese women is more often associated with markers of gonadotropin dysfunction, such as elevated LH and LH/FSH ratios, than in obese women with PCOS [43]. High LH and LH/FSH imbalances are involved in ovulation disturbances in PCOS. LH also increases androgen levels by participating in AND production [44]. In addition, the diurnal changes in the circulating concentrations of LH and insulin in PCOS women follow a similar time course [45]. Roshni et al. revealed that PIO and MET treatment altered the mRNA expression of LH receptors and FSH receptors in PCOS rats [46]. Nestler et al. observed downregulation of mean serum LH levels after 6 weeks of MET therapy (1500 mg/day) in normal-weight and overweight women with PCOS [36]. Rodolfo et al. found that postnatal rosiglitazone treatment completely restored GnRH-stimulated LH pulse peaks in a sheep model of PCOS [47]. Ali et al. found that in the overweight and obese PCOS population (BMI ≥ 25 kg/m^2^), PIOMET treatment (30 mg/d for PIO and 1000 mg/d for MET) for 3 months effectively reduced LH levels in women with PCOS compared with MET monotherapy (1000 mg/d) [38]. We observed a significant decrease in LH levels with MET at 12 weeks of treatment, which is consistent with the findings of Nestler et al. This suggests that the improvement in LH levels with MET is independent of body weight. In addition, we also observed that LH levels and LH/FSH significantly improved after 4 weeks of PIOMET treatment, suggesting that PIO may be involved in the control of GnRH secretion via hypothalamic neural pathways.

Some patients with PCOS have a characteristic polycystic ovary appearance. AMH is secreted by granulosa cells in secondary follicles and acts independently of the hypothalamic-pituitary-ovarian axis in the early stage of follicular development [4]. AMH can be used to assess the number of growing follicles in the ovary and the ovarian reserve function. It is more sensitive than ultrasound for reflecting follicles smaller than 2 mm. It is also not affected by the menstrual cycle [48, 49]. Previous studies have reported conflicting results regarding the improvement of AMH levels by MET [50–52]. Animal studies have shown that PIO can reduce serum AMH levels, the total number of atretic follicles, and atretic follicle rates in obese PCOS rats [53, 54]. Selenay et al. observed that MET therapy did not improve serum AMH levels in rats with dehydroepiandrosterone-induced PCOS [55]. Soldat-Stankovid et al. observed a decrease in AMH levels in normal-weight patients with PCOS (BMI ≤ 25 kg/m^2^) treated with MET (1500 mg/d) for 6 months [51]. We also found that AMH levels in both groups showed a significant improvement after 12 weeks of treatment; Moreover, the PIOMET group was significantly better than MET monotherapy, suggesting that PIO may be effective in improving ovulation and restoring ovarian function by regulating AMH involvement in follicular development.

Metabolic abnormalities often occur with obesity; however, normal-weight women with PCOS also have a variety of metabolic abnormalities [4]. Studies on metabolic correlates in patients with PCOS have shown that compared with BMI-matched healthy controls, plasma insulin, low-density lipoprotein, total cholesterol, and plasma gamma glutamyl transferase levels were significantly higher in normal-weight women with PCOS [4]. MET increases hepatic glucose output to improve insulin sensitivity in peripheral tissues, and TZDs directly activate PPAR-γ receptors in peripheral tissues to exert insulin-sensitizing effects [14]. Yang et al. demonstrated a significant decrease in FINS and HOMA-IR after 3–12 months of treatment with MET (500–1500 mg/d) in an overweight/obese PCOS group (BMI ≥ 25 kg/m^2^), whereas no such change was observed in the non-obese group (BMI < 25 kg/m^2^) [34]. Ortega et al. showed that in an overweight/obese population with PCOS (BMI ≥ 25 kg/m^2^), fasting serum insulin concentration and AUCIns during a 2-h oral glucose tolerance test decreased after 6 months of MET (2550 mg/d) versus PIO (30 mg/d) treatment, with no significant difference between the two groups [56]. Tan et al. observed significant decreases in FPG, FINS, and HOMA-IR after 6 months of treatment with MET (1500 mg/d) or rosiglitazone (4 mg/d) in normal-weight women with PCOS (BMI < 25 kg/m^2^) compared to the respective pre-treatment periods, with lower FINS levels and HOMA-IR in the rosiglitazone group; however, the differences were not significant compared to the MET group [32]. Our study observed that after 12 weeks of treatment, FPG, FINS, and HOMA-IR showed a decreasing trend from the baseline between the two groups; however, no significant difference was observed. Although the AUC curve of the OGIRT in the PIOMET group showed a downward trend in glucose and insulin areas from baseline, OGIRT only at 120 min showed a significant decrease in glucose and insulin from baseline, whereas no such trend was found in the MET group. In addition, blood glucose levels decreased more significantly at 120 and 180 min of OGIRT in the PIOMET group than in the MET group, suggesting a possible advantage of PIOMET in improving metabolism in normal-weight women with PCOS. Since previous studies on metabolic changes after insulin sensitization in normal-weight women with PCOS are scarce and the number and duration of interventions limit the comparison of statistical differences, further confirmation is needed in future studies with large sample sizes.

Previous studies have shown that in obese patients with PCOS, MET treatment can reduce body weight, whereas PIO treatment can further aggravate obesity [56]. Yang et al. demonstrated a reduction in BMI after 6 months of MET (1500 mg/d) treatment in both overweight and normal-weight women with PCOS (with a BMI cut-off of 25 kg/m^2^) [34]. Guo et al. did not observe any significant weight change in either group after 12 weeks of MET (1500 mg/d) or PIOMET (45 mg for PIO and 1500 mg/d for MET) treatment in patients with PCOS with unrestricted BMI [37]. Ali et al. revealed that 12 weeks of MET (1000 mg/d) and PIOMET (30 mg/d for PIO and 1000 mg/d for MET) treatment resulted in weight loss in the PCOS population (most had BMI ≥ 25 kg/m^2^) [38]. Our study found that after 12 weeks of treatment, the MET group lost significant weight from baseline, and the PIOMET weight did not change, with no significant differences between the two groups. This was possibly due to the small number of participants or the fact that the initial BMI of our Chinese participants was lower than that of the European participants included by Ali et al. [38]. In addition, no serious gastrointestinal discomfort was observed in normal-weight women with PCOS throughout the treatment with low-dose MET (1000 mg/d), and no adverse events occurred with full PIOMET treatment, which is also consistent with the study by Zeng et al. [57].

This study determined the effect of PIOMET on improving menstruation, sex hormone levels, and glucose metabolism in normal-weight patients with PCOS. We excluded the influence of obesity on the effect of PIOMET on the reproductive, hyperandrogenic, and metabolic status of PCOS. PIOMET fixed-dose combination therapy is convenient, inexpensive, and well-tolerated, increasing patient compliance. However, owing to the impact of the novel coronavirus pandemic, the small sample size and high dropout rate are the main limitations of this study, although the degree of statistical significance can be determined by calculating the sample size. Further extensive sample studies are needed to confirm these conclusions. Second, it is necessary to observe other reproductive endocrine and metabolic indicators, such as glycated hemoglobin, lipid profiles, hirsutism scores, and results from vaginal ultrasound, to better substantiate the effects of three months of MET monotherapy and combined treatment with PIO on this syndrome. Third, this is an open-label, single-center, randomized controlled trial, where factors such as lifestyle management during treatment, energy intake, and potential biases arising from subjective patient assessments and recall deviations can introduce information bias and confounding bias into the experiment. Additionally, although we observed that PIOMET therapy was more effective than MET monotherapy in many aspects, PIO monotherapy was not included in this study; therefore, we could not determine whether the therapeutic effect of PIOMET was the effect of PIO monotherapy or the synergistic effect of PIO and MET. Several clinical studies on PIO monotherapy are required to investigate the efficacy of PIO, MET, and PIOMET therapies in treating normal-weight women with PCOS. In addition, we conducted the study intervention in normal-weight women with PCOS for only 3 months, and there may be an additional benefit of extending the duration of treatment in this population. Furthermore, we defined normal weight as 18.5 ≤ BMI < 25 kg/m^2^, according to the World Health Organization. As all participants included in this research were Chinese women with PCOS, further exploration is needed to ascertain the adaptability of the study conclusions within the context of the normal weight standards specific to China. Summarily, this study provides insights on the clinical treatment of this population and further clues for a future in-depth study of the clinical treatment of normal-weight women with PCOS.

In conclusion, PIOMET treatment may have more benefits in improving SHBG, AMH, and postprandial glucose levels than MET monotherapy in normal-weight women with PCOS, and did not affect weight. However, these results need to be confirmed in larger study populations.

## Data Availability

The raw data supporting the conclusions of this article will be made available by the authors, without undue reservation.

## Abbreviations

PCOS: polycystic ovary syndrome
IR: insulin resistance
HA: hyperinsulinaemia
LH: luteinizing hormone
FSH: follicle-stimulating hormone
TT: total testosterone
FAI: free androgen index
SHBG: sex hormone-binding globulin
AND: androstenedione
DHEAS: dehydroepiandrosterone sulfate
AMH: anti-Mullerian hormone
BMI: body mass index
FPG: fasting plasma glucose
FINS: fasting insulin
AUCGlu: area under the glucose curve
AUCIns: area under the insulin curve
HOMA-IR: homoeostasis model assessment of insulin resistance
PIOMET: pioglitazone hydrochloride and metformin hydrochloride tablets
MET: metformin
TZDs: thiazolidinediones
PPAR-γ: peroxisomal proliferating-activated receptor-γ
T2DM: type 2 diabetes
